# Supplementary material for: South Asian, Black and White ethnicity and the effect of potentially modifiable risk factors for dementia: A study in English electronic health records
Source: PLoS One. 2023 Oct 11;18(10):e0289893. doi: 10.1371/journal.pone.0289893 (PMC10566703; doi:10.1371/journal.pone.0289893)
Supplement: S1 File — (DOCX) [file pone.0289893.s002.docx]

# Supplementary material – dementia risk factors and ethnicity

## Definitions and validation of risk factors and other variables

Midlife hypertension – A diagnosis of midlife hypertension was assigned if a patient had at least two of the following criteria occurring before age 65 in their health record as combinations of diagnostic codes and medications/BP measures increase accuracy and better reflect national prevalence data^1^: i) hypertension diagnostic code in CPRD or HES, ii) blood pressure reading of ≥140mmHg systolic or ≥90mmHg diastolic, iii) at least two prescriptions of antihypertensive medications (BNF chapters 2.2.1, 2.2.3, 2.2.4, 2.4, 2.5 and 2.6.2). Date of hypertension was the earliest of any of the diagnostic or medication codes, at any point in the person’s records.

Diabetes – A diagnosis of diabetes was assigned if a patient, at any age, had at least two of the following criteria at any point in their health record, as outlined in a previously validated algorithm^2^: i) diagnostic code for diabetes or diabetes complications in CPRD or HES, ii) prescription of diabetes medication (BNF chapters 6.1.1 or 6.1.2), iii) HbA1c >48 mmol/mol or 6.5%, iv) plasma glucose >11.1 mmol/l, v) fasting plasma glucose>7.0 mmol/l, vi) post-prandial glucose>11.1mmol/l, or v) abnormal glucose tolerance test (>11mmol/l after 2 hours).

Excess alcohol – Any patient with a Read code in CPRD indicating binge or excess alcohol consumption, or records indicating consumption of ≥14 units’ alcohol per week for women and ≥21 units per week for men was classified as drinking excess alcohol, using codes closest to the start date of the cohort.

Midlife obesity - Participants were classified as obese if they had any of the following recorded prior to age 65 in their records: i) diagnostic code in CPRD or HES indicating obesity, ii) prescription of anti-obesity medications (BNF sections 4.5.1 or 4.5.2), iii) referral for or record of bariatric surgery, iv) recorded BMI of ≥30kg/m^2^. Date of diagnosis was the earliest recorded diagnostic or medication/BMI code meeting criteria.

Smoking – participants were classified as current or non-current smokers^3^ based on coding in CPRD and HES, using codes closest to the start date of the cohort.

Depression – Participants were classified as having a history of depression if they had any diagnostic codes for depression or depressive symptoms in CPRD or ICD-10 codes for depressive disorder in HES at any point in their adult life. Primary care Read code lists have been found to be valid against clinician record review and sensitivity is improved by including historical diagnoses of depression^4^. Read code lists for current or previous depression are as used in previous papers^5^.

Lipids – Participants were classified as having high cholesterol if they had a recorded total cholesterol of 5mmol/l in CPRD at any time, were defined as having dyslipidaemia if they had a diagnostic code indicating dyslipidaemia either in CPRD or HES. The earliest date of any of these was used as the date of exposure. We also investigated association of dementia with HDL and LDL cholesterol specifically using clinical definitions of normal ranges for these (≥1mmol/l for HDL and ≤3mmol/l for LDL)^6^. We did not include prescriptions of statins or other cholesterol lowering medications as part of our criteria as these are often used to manage cardiovascular risk in the absence of high cholesterol^7^.

Sleep disturbance - We searched for Read codes (in CPRD) and ICD-10 codes (in HES) relating to sleep disorders at any age. We excluded disorders relating to specific medical disorders like sleeping sickness or sleep disorders secondary to drugs or alcohol. We excluded conditions such as narcolepsy, parasomnias and sleep movement disorders but included any terms relating to poor sleep quality, insomnia and sleep disordered breathing as these have evidence from meta-analysis of an association with dementia and cognitive impairment ^8^. We classified them into categories based on the International Classification of Sleep Disorders-Third Edition^9^ produced by the American Academy of Sleep Medicine. We also included patients who had more than 2 weeks’ prescription of hypnotics (BNF chapter 4.1.1) prescribed in a year at any time in their health records as we reasoned this was indicative of difficulty in sleeping^10^.

Hearing loss – Participants were defined as having hearing loss if they had Read (in CPRD) or ICD-10 codes (in HES) for sensorineural/central hearing loss or mixed hearing loss or presbycusis, not purely conductive hearing loss, at any age. We excluded those with congenital hearing loss as the evidence is for those with acquired hearing loss. We excluded those with deafness as part of a specified medical syndrome, those with trauma or drug-related deafness and those who were classified as deaf-blind as these were all likely to be associated with multiple confounding factors. We separated into those with and without a record of hearing aids in their records but bone-anchored hearing aids were not included as these are for specific forms of hearing loss, usually with congenital malformations or conductive hearing loss. We included referrals to audiology services and audiometric testing as signs of possible hearing loss.

Traumatic brain injury – We searched for Read codes (in CPRD) or ICD-10 codes (in HES) relating to brain injury in all adults. These included “concussion”, “cerebral contusion”, “head injury”, “brain injury”, “intracranial injury”, head or skull fracture, subarachnoid haemorrhage, extradural and subdural haemorrhage related to injury. We excluded intracerebral haemorrhage if trauma was not specified as a cause. ICD-10 defines mild traumatic brain injury (TBI) as concussion and severe TBI as skull fracture, oedema, brain injury or bleed.

Code lists for hearing loss, traumatic brain injury and sleep disorders had not been previously validated, so for these variables, a clinician compiled a list of clinical and medicine codes and an independent clinician checked a minimum of a random 10% of all Read codes and ICD-10 codes and any disagreements about categorisation of disorders was resolved by discussion.

## Charlson index

The original Charlson comorbidity index is a validated way of measuring medical comorbidity^11^. It is calculated by adding up the number of comorbidities and giving additional points for every 10 years a person is over the age of 50. Conditions includes myocardial infarction, heart failure, diabetes mellitus, stroke, arterial disease, cancer, AIDS, liver disease, connective tissue disorders, peptic ulcers, metastatic cancer and dementia. As our regression models adjusted for age we excluded this from our comorbidity index. We also did not include diabetes and dementia as these were a risk factor and outcome respectively. We searched electronic records for Read codes indicating all of the other medical conditions and assigned a point for any indication of any of the conditions if they occurred at any point in the person’s medical records. We summed up all disease indicators to give a total score on the modified Charlson index. This was treated as a continuous variable in regression models.

## Additional analyses

| **Risk factors** | **Main effects** | | | **Interaction terms** | | | |
| --- | --- | --- | --- | --- | --- | --- | --- |
|  |  |  |  | **South Asian** | | **Black** | |
|  | **HR** | **LCL** | **UCL** | **HR** | **p value** | **HR** | **p value** |
| **Hypertension** | 1.30 | 1.27 | 1.33 | 2.19 | <0.0001 | 1.95 | <0.0001 |
| **Obesity** | 1.45 | 1.41 | 1.50 | 1.50 | 0.0002 | 1.32 | 0.003 |
| **Hearing loss** | 1.26 | 1.21 | 1.31 | 1.60 | 0.001 | 1.50 | 0.01 |
| **Smoker** | 1.17 | 1.14 | 1.21 | 1.34 | 0.008 | 1.38 | 0.003 |
| **Excess alcohol** | 1.15 | 1.10 | 1.20 | 1.14 | 0.61 | 0.77 | 0.37 |
| **Diabetes** | 1.28 | 1.24 | 1.34 | 2.21 | <0.0001 | 1.87 | <0.0001 |
| **Dyslipidaemia** | 1.65 | 1.61 | 1.69 | 1.30 | 0.006 | 1.45 | 0.0002 |
| **Low HDL** | 1.49 | 1.40 | 1.57 | 1.83 | <0.0001 | 1.22 | 0.30 |
| **High LDL** | 1.64 | 1.58 | 1.70 | 1.13 | 0.15 | 1.28 | 0.01 |
| **Depression** | 1.70 | 1.62 | 1.77 | 1.41 | 0.03 | 1.25 | 0.18 |
| **Sleep disorder** | 1.08 | 1.05 | 1.10 | 1.46 | <0.0001 | 0.99 | 0.95 |
| **Brain injury** | 1.48 | 1.39 | 1.58 | 1.25 | 0.49 | 1.03 | 0.94 |

**Table S1: Competing risks analysis, using multiply imputed data, adjusted for age, sex and Index of Multiple Deprivation (IMD). LCL = lower 95% confidence limit, UCL = upper 95% confidence limit**

| **Risk factors** | **Main effects** | | | **Interaction terms** | | | |
| --- | --- | --- | --- | --- | --- | --- | --- |
|  |  |  |  | **South Asian** | | **Black** | |
|  | **HR** | **LCL** | **UCL** | **HR** | **p value** | **HR** | **p value** |
| **Hypertension** | 1.11 | 1.10 | 1.13 | 1.18 | 0.12 | 0.83 | 0.09 |
| **Obesity** | 1.34 | 1.32 | 1.37 | 1.14 | 0.29 | 0.88 | 0.23 |
| **Hearing loss** | 1.22 | 1.19 | 1.24 | 1.28 | 0.02 | 1.14 | 0.40 |
| **Smoker** | 1.12 | 1.10 | 1.14 | 1.09 | 0.42 | 1.01 | 0.96 |
| **Excess alcohol** | 1.09 | 1.06 | 1.12 | 0.95 | 0.86 | 0.58 | 0.08 |
| **Diabetes** | 1.19 | 1.16 | 1.21 | 1.51 | <0.0001 | 1.15 | 0.16 |
| **Dyslipidaemia** | 1.52 | 1.50 | 1.54 | 0.92 | 0.41 | 0.91 | 0.34 |
| **Low HDL** | 1.34 | 1.30 | 1.38 | 1.31 | 0.04 | 0.92 | 0.60 |
| **High LDL** | 1.53 | 1.50 | 1.56 | 0.84 | 0.12 | 0.85 | 0.10 |
| **Depression** | 1.59 | 1.55 | 1.62 | 1.07 | 0.68 | 0.94 | 0.76 |
| **Sleep disorder** | 1.04 | 1.02 | 1.05 | 1.24 | 0.05 | 0.88 | 0.27 |
| **Brain injury** | 1.44 | 1.38 | 1.49 | 0.93 | 0.82 | 0.74 | 0.37 |

**Table S2 - competing risks regression using complete case sample and adjusting for Charlson index as well as age, sex and Index of Multiple Deprivation (IMD)**. **LCL= lower 95% confidence limit, UCL = upper 95% confidence limit**

1. Peng M, Chen G, Kaplan GG, et al. Methods of defining hypertension in electronic medical records: validation against national survey data. *Journal of Public Health* 2016; **38**(3): e392-e9.

2. Sharma M, Nazareth I, Petersen I. Trends in incidence, prevalence and prescribing in type 2 diabetes mellitus between 2000 and 2013 in primary care: a retrospective cohort study. *BMJ open* 2016; **6**(1).

3. Marston L, Carpenter JR, Walters KR, et al. Smoker, ex-smoker or non-smoker? The validity of routinely recorded smoking status in UK primary care: a cross-sectional study. *BMJ open* 2014; **4**(4): e004958.

4. John A, McGregor J, Fone D, et al. Case-finding for common mental disorders of anxiety and depression in primary care: an external validation of routinely collected data. *BMC medical informatics and decision making* 2016; **16**(1): 35.

5. Rait G, Walters K, Griffin M, Buszewicz M, Petersen I, Nazareth I. Recent trends in the incidence of recorded depression in primary care. *The British Journal of Psychiatry* 2009; **195**(6): 520-4.

6. Service NH. High cholesterol. 2020. <https://www.nhs.uk/conditions/high-cholesterol/> (accessed 14/07/2021 2021).

7. Service NH. Statins. 2020. <https://www.nhs.uk/conditions/statins/> (accessed 14/07/2021 2021).

8. Bubu OM, Brannick M, Mortimer J, et al. Sleep, cognitive impairment, and Alzheimer’s disease: a systematic review and meta-analysis. *Sleep* 2017; **40**(1): zsw032.

9. Sateia MJ. International classification of sleep disorders. *Chest* 2014; **146**(5): 1387-94.

10. Service NH. Insomnia. 2020. <https://www.nhs.uk/conditions/insomnia/> (accessed 14/07/2021 2021).

11. Charlson ME, Pompei P, Ales KL, MacKenzie CR. A new method of classifying prognostic comorbidity in longitudinal studies: development and validation. *Journal of Clinical Epidemiology* 1987; **40**(5): 373-83.
